# Unleashing Tumour-Dendritic Cells to Fight Cancer by Tackling Their Three A’s: Abundance, Activation and Antigen-Delivery

**DOI:** 10.3390/cancers11050670

**Published:** 2019-05-14

**Authors:** Aleksandar Murgaski, Pauline M. R. Bardet, Sana M. Arnouk, Emile J. Clappaert, Damya Laoui

**Affiliations:** 1 Myeloid Cell Immunology Lab, VIB Center for Inflammation Research, 1050 Brussels, Belgium; murgaski.aleksandar@vub.be (A.M.); pauline.bardet@vub.be (P.M.R.B.); sana.arnouk@vub.be (S.M.A.); emile.clappaert@vub.be (E.J.C.); 2 Lab of Cellular and Molecular Immunology, Vrije Universiteit Brussel, 1050 Brussels, Belgium

**Keywords:** dendritic cells, tumour-associated dendritic cells, immunotherapy, DC-therapy, DC-vaccinations

## Abstract

Recent advances in cancer immunotherapy have mainly focused on re-activating T-cell responses against cancer cells. However, both priming and activation of effector T-cell responses against cancer-specific antigens require cross-talk with dendritic cells (DCs), which are responsible for the capturing, processing and presentation of tumour-(neo)antigens to T cells. DCs consequently constitute an essential target in efforts to generate therapeutic immunity against cancer. This review will discuss recent research that is unlocking the cancer-fighting potential of tumour-infiltrating DCs. First, the complexity of DCs in the tumour microenvironment regarding the different subsets and the difficulty of translating mouse data into equivalent human data will be briefly touched upon. Mainly, possible solutions to problems currently faced in DC-based cancer treatments will be discussed, including their infiltration into tumours, activation strategies, and antigen delivery methods. In this way, we hope to put together a broad picture of potential synergistic therapies that could be implemented to harness the full capacity of tumour-infiltrating DCs to stimulate anti-tumour immune responses in patients.

## 1. Introduction

It is becoming increasingly apparent that our immune system is capable of fighting cancer. Understanding the interplay between our immune system and cancer has led to the development of new treatments that can prolong survival in once-thought terminal patients. The success that immune checkpoint inhibitors (ICIs) have had in the clinic has sparked renewed interest and investment in the tumour immunology field [1]. However, durable responses to immunotherapy are only seen in a minority of patients [2,3,4]. A common trait among many treatment responsive patients is a high neoantigen load; a characteristic which often correlates with a strong adaptive immune response against the tumour [5]. This response is required for the ICIs to release the brakes that the tumour places on the immune system.

On the other hand, patients who do present a high neoantigen load may not respond to ICIs due to an immunosuppressive tumour microenvironment (TME) [6]. In these patients, anti-tumour immune responses are shut down by immunosuppressive cells such as tumour-associated macrophages (TAMs) and myeloid-derived suppressor cells (MDSCs). Interactions between these suppressive cells and effector T cells can lead to T-cell exhaustion, a state of T-cell dysfunction seen during chronic inflammation [7]. While ICIs can rescue some T cells from these interactions, suppression of the TME might still be too strong for T cells to fully overcome this obstacle, resulting in continued tumour progression. Therefore, it is imperative to improve the adaptive immune response against the tumour while simultaneously redirecting the TME toward a more immuno-permissive state. In this light, harnessing the potential of dendritic cells (DCs) that reside within tumours is one avenue of research that could yield positive clinical results for cancer patients in the near future [8].

It is well established that DCs have the ability to link both the innate and adaptive immune systems and to initiate immune responses [9]. In the age of immunotherapy, this capacity to generate adaptive immune responses is considered to be imperative. DCs can activate T-cell responses with great efficacy due to their high expression of co-stimulatory molecules and specific T-cell adhesion molecules. However, DCs are also capable of shutting down immune responses by expressing high levels of co-inhibitory molecules [10]. Therefore, understanding and exploiting mechanisms relating to the function of tumour-associated DCs (TADCs) can lead to the development of powerful tools to fight cancer.

## 2. DC Subsets: Adding Another Layer of Complexity to DC-Based Therapies

The human body can contain at least four different subsets of DCs [11], with each of these having a murine equivalent [12]. Moreover, all of these subsets have been shown to reside in tumoural tissue [13]. Plasmacytoid DCs (pDCs) are CD11c^low^, MHC-II^+^, B220^+^, Siglec-H^+^ in mice, and represent the front line of anti-viral immune responses due to their specialised type-I interferon secretion [14]. In humans, these pDCs can be identified as CD123^+^CD33^−^ [15,16]. The two distinct subsets of conventional dendritic cells (cDCs), namely cDC1 and cDC2, both develop from committed bone marrow progenitor cells [17]. They are specialised in stimulating cytotoxic T lymphocyte (CTL) responses and T helper 17 (Th17), or Th2 responses, respectively. In mice, cDC1s have a high expression of CD11c, MHC-II, XCR1, Clec9A, IRF8, and depending on the organ also CD103 or CD8α. In humans, they correspond to the BDCA-3^+^ subset. cDC2s are more difficult to identify due to the current lack of a canonical marker, but can be classified based on their higher expression of CD11c, MHC-II, CD172a, IRF4 and CD11b [18]. In humans, this subset is characterised by the expression of CD1c (BDCA-1) [18]. During inflammatory settings such as in a tumour context, a fourth kind of DC can be identified, being monocyte-derived DCs (Mo-DCs). Mo-DCs are CD11c^+^, MHC-II^+^, F4/80^−^, CD64^+^ in mice, CD14^+^ and in some cases CD16^+^ in humans. These cells are closely related to macrophages and have been shown to display similarly high plasticity [19]. In the TME, Mo-DCs were shown to exhibit an immunosuppressive phenotype promoting tumour progression [13,20].

Obtaining human tissue samples is challenging. Therefore, the bulk of human DC research is performed using blood-derived cDCs, of which some studies have suggested they are precursor cells that have yet to fully differentiate [21,22]. Aligning mouse and human DC knowledge has shown that there are considerable differences between the two species. Nevertheless, DC subsets between human and mouse also share certain similarities [23]. The cDC subsets show strong reliance on Flt3L for their development, regardless of the studied species [24]. cDC1 homology has been shown in human and mouse through comparative transcriptomics, receptor expression, transcription factors, cytokine secretions, and functional assays [25,26,27]. cDC2 in both human and mouse are capable of stimulating Th17 responses and depend on IRF4 for their differentiation [28]. Nevertheless, human cDCs differ from mouse cDCs. For example, the transcription factor IRF8 is only critical for mouse cDC1s; however, patients that have disease-causing mutations affecting IRF8 lack both cDC subsets [29]. It has been shown that in humans both cDC subsets can cross-present antigen with similar efficiency, while in mice this role is mainly attributed to cDC1s [30,31]. Activated blood cDC2s from humans secrete higher levels of IL-12, leading to higher cytotoxic molecule production by CTLs than cDC1, a role mouse cDC2 cannot perform [32]. With these differences in mind, any research that is being translated from mice to humans should take extra care to ensure its functional consistency between species.

Due to the existence of these different DC subsets, each embodying different functions, DC abundance in tumours has been correlated with both positive and negative clinical outcomes [33,34]. As such, a high infiltration of mature, active cDCs in tumours, correlates with a strong immune activation and recruitment of anti-tumour immune cells such as CTLs [35]. However, in many cases, the TME is able to suppress DC function [36]. Hence, by understanding how the TME shapes DC activation status and DC functionality, rationales for combinations of therapies can be formulated to unlock the full anti-tumour potential of TADCs. Besides influencing tumour-resident DCs, the tumour affects the level and function of circulating DCs. Indeed, in several cancer types, including melanoma and colorectal cancer, late stage patients show decreased levels and functional impairment of distinct DC subsets in the peripheral blood [37,38,39,40,41].

The future of cancer therapy lies in combinatorial treatments. A multi-faceted approach that targets a series of different pathways will likely address several of the issues regarding TADC-mediated responses such as (i) the paucity or low immunogenicity of known tumour-antigens, (ii) the inefficient uptake and presentation of tumour-antigens by DCs, (iii) the low abundance of TADCs in the tumour and (iv) the immunosuppressive TME, which limits T-cell infiltration and effector functions [7,8].

This review focusses on DCs in the TME, aiming at providing a broader view of the potential combinations of treatments that could work synergistically to activate TADCs, which can in turn stimulate long-lasting anti-tumour responses in patients.

## 3. Abundance: Increasing DC Infiltration into Tumours

Murine studies have shown that during early tumour development DCs play important anti-tumoural roles, and their depletion, using CD11c-DTR transgenic mice, resulted in faster tumour growth [42]. Hence, increasing the infiltration of DCs into tumours during the early growth phase could potentially inhibit tumour growth. On the other hand, DCs infiltrating late stage tumours were shown to have an immature tolerogenic state. Depleting these DCs during later stages of tumour growth resulted in reduced tumour growth, suggesting a key phenotype switch in the TADC population [42]. This illustrates the importance of the factors found within the TME in skewing the phenotype of tumour-infiltrating immune cells.

Tumour-infiltrating cDCs are scarce, yet the presence of mature cDCs correlates with favourable immune infiltration and an improved prognosis in multiple cancer types [43]. The presence of cDC1s within tumours has also been shown to correlate positively with the outcome as well as responsiveness to anti-PD-1 ICI therapy [44,45]. Moreover, anti-PD-1 efficacy was shown to depend on the crosstalk between T cells and IL-12-producing cDCs [46]. This being the case, any treatment attempting to harness tumour-infiltrating cDCs must address the issue of their low relative abundance.

Mechanisms used to increase DC infiltration into tumours involve the administration of different cytokines. Flt3L has been shown to be required during multiple stages of DC development, from bone marrow progenitors to their tissue-homing [16,47]. In a study using the B16 melanoma mouse model, Salmon et al. treated tumour-bearing mice with Flt3L and observed an increase in the amount of CD103^+^ DCs infiltrating the tumour. While Flt3L administration alone would slow tumour growth, combining Flt3L treatment with Poly I:C, a Toll-like receptor 3 (TLR3) ligand, induced a much more dramatic B16 tumour regression. The administration of Poly I:C matured tumour-infiltrating DCs that were initially in a more immature state and expressed less CD40, CD86 and MHCII, hence eliciting a greater anti-tumour activity [48]. Granulocyte-macrophage colony-stimulating factor (GM-CSF) is another important cytokine in terms of DC development and migration [49]. GM-CSF was incorporated into one of the earliest developed cancer vaccines, GVAX [50]. This treatment genetically modifies whole cancer cells, causing them to secrete GM-CSF. These cells are irradiated to arrest their proliferation and then subsequently administered to patients, with the aim of attracting and activating DCs in order to stimulate antigen specific responses. Beneficial results were observed during a phase II trial of pancreatic cancer patients [51]; however, a phase IIb trial showed no difference between the cyclophosphamide/GVAX/CRS-207-treated group and the control chemotherapy group [52]. Flt3L and GM-CSF have been the two most commonly used treatments to increase DC abundance. Nevertheless, recent research is unravelling other potential DC migratory signals. NK cells are being recognised as an important immune cell that can guide cDC migration due to their Flt3L secretion [45]. The recruitment of cDC1s to murine tumours was shown to depend on other NK cell-derived chemoattractants, specifically XCL1 and CCL5. Prostaglandin E2 (PGE_2_) from the TME impaired NK-cell chemokine production, thus enabling tumour immune escape [53].

One group inserted XCL1 and soluble Flt3L into a Semliki Forest Virus vector, which was then injected intratumourally, resulting in delayed tumour growth of MC38 and B16-OVA murine tumours [54]. Depending on the tumour model used, added beneficial effects were observed when these tumours were concurrently treated with either anti-PD-1, anti-CD137 or anti-CTLA-4. These effects were not seen in Batf3-deficient mice, suggesting that cross-priming of tumour antigens by Batf3-dependent DCs is crucial to the efficacy of immuno-stimulatory monoclonal antibodies (mAbs) [54]. Other research demonstrated that the use of DPP4, which inhibits CXCL9/CXCL10 processing intratumourally, increased cDC1 infiltration into B16F10 murine melanoma tumours [55]. By maintaining high CXCL9/CXCL10 levels within the tumour, these ligands could interact with their cognate receptor, CXCR3, which was observed to be expressed higher on pre-cDC1 in the blood than on other DCs subsets. While increasing the factors that guide TADC recruitment has yielded promising results, the inhibition of cytokines that suppress TADC migration should also be explored. For instance, human pancreatic and breast tumours secrete factors that suppress cDC1 development in the bone marrow. This effect was determined to be the result of granulocyte-colony-stimulating factor (G-CSF) being secreted by the tumour, which inhibited cDC1 development by impairing IRF8 expression in cDC progenitors. Neutralising G-CSF by using anti-G-CSF IgGs recovered IRF8 expression and pre-cDC levels in the bone marrow and blood [56]. Targeting interactions that either reduce or increase DC recruitment to tumours should be considered when attempting to develop any robust TADC-based cancer treatment.

Another option to increase DC levels within tumours is to directly administer DCs to patients (or mice), a technique which is reviewed in Aznar et al., specifically focussing on intratumoural immunotherapy [57]. While this technique can yield beneficial results, there are many variables to take into consideration when injecting DCs, one of the most important being the route of administration. The envisioned target of the DCs has an influence over where the DCs should be injected. It has been shown that murine CXCL10-producing CD103^+^ DCs are important in effector T-cell navigation to the tumour, and when these cells are no longer present in the TME, an efficient anti-tumour immunity is lacking [58]. Conversely, another study showed that depletion of cDCs using Zbtb46-DTR mice did not alter the intratumoural CTL levels. However, CTL proliferation and expansion in the tumour-draining lymph nodes (tdLNs) was impaired, indicating an important role for cDCs in tdLNs [59]. It is therefore important to understand the possible destinations of DCs and how these destinations vary between administration routes. In vivo fluorescent imaging allows for the tracking of DC trafficking and the comparison between various delivery routes [60]. Trafficking of DCs to the tumour was shown to happen when mice were injected intravenously (IV), subcutaneously (SC), or intratumourally (IT). While all injection routes delivered DCs to tumours, they varied based on the other locations DCs could migrate to. SC injections showed the best potential to get DCs to LNs. Meanwhile, IV injections resulted in DC migration to the lung, intestines and spleen. Additionally, it is important to fully characterize the nature and/or activation state of the type of DC that is being administered to patients (or mice). A highly plastic DC, such as a Mo-DC, can be easily skewed towards a pro-tumoural phenotype, defeating the purpose of the injection. As such, a population coexpressing both the DC marker BDCA1 and the monocyte marker CD14, was found to be upregulated in the circulation of melanoma patients and displayed an immunosuppressive potential [20]. Therefore, it is imperative that the correct DC subset is used for the envisioned scenario, with BDCA-3^+^ or BDCA-1^+^ CD14^−^ DCs being the most likely candidates, as these DCs are much more committed to their phenotype [13].

## 4. Activation: Maturing DCs Residing within Tumours

**Figure 1:**
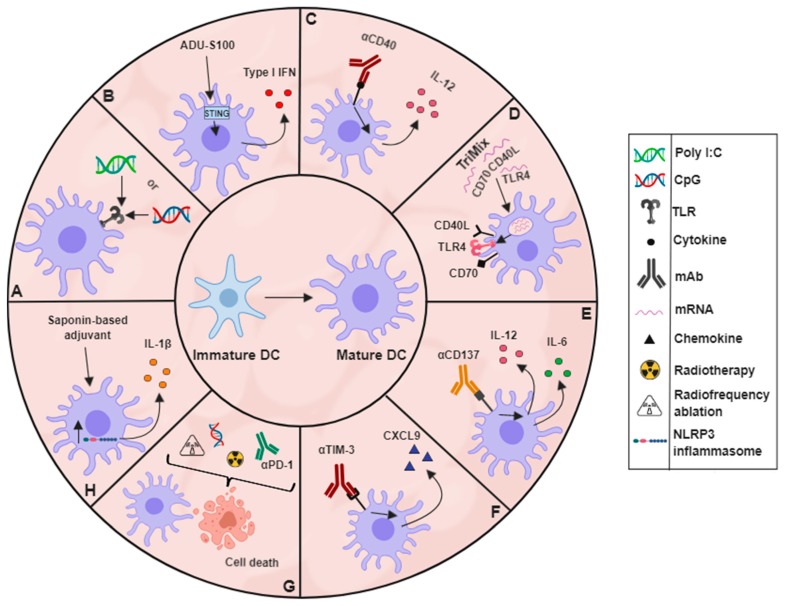
Different strategies with the potential to activate DCs within the TME. (**A**) TLR agonist-mediated DC stimulation via CpG or Poly I:C. (**B**) Activation of STING via STING agonists. (**C**) Direct maturation via CD40 agonism. (**D**) TriMix mRNA administration. (**E**) Altering the DC phenotype by using an agonistic mAb to CD137 (**E**) or an antagonistic antibody to TIM-3 (**F**). (**G**) Different combination therapies causing immunogenic cell death while subsequently activating DCs. (**H**) Use of saponin-based adjuvants to activate DCs.

Any DC-based treatment should take into account the activation and maturation state of TADCs due to this being an important factor in tumour progression and response to therapies [61]. As was observed by Salmon et al., expansion of murine cDCs by Flt3L treatment led to an accumulation of cDCs with an immature phenotype in the TME [48]. Stimulation of these immature cDCs can lead to strong anti-tumour effects. However, not all methods aiming to activate DCs result in the same strength of response or maturation [62]. There are many potential adjuvants being investigated for their ability to stimulate DCs within the TME (Figure 1). These include different TLR agonists, stimulatory antibody treatments, radiotherapy, chemotherapy and cryotherapy, among others. All of these treatments aim to increase the expression of co-stimulatory molecules on and cytokine production in DCs within the tumour, allowing them to better activate CTLs within the TME and lymphoid organs.

The multitude of TLR agonists that are being investigated within the scope of cancer treatments have warranted their own reviews [63,64,65,66,67]. Therefore, this review will only briefly touch on their action and applicability in the tumour scenario. Of the different ligands capable of stimulating TLRs, two of the most commonly used are CpG DNA and Poly I:C. CpG DNA has been used for many years in animal models and has been incorporated into the clinic with treatment usually being well tolerated (Figure 1A) [68]. Intratumoural CpG DNA injection resulted in tumour growth inhibition, mediated by a massive infiltration of activated neutrophils which contributed to tumour-associated cDC activation and the consequential anti-tumour T-cell response in murine EG7-OVA tumours [69]. Of note, results gathered from experiments performed using transplantable tumour models expressing an artificial surrogate tumour antigen such as ovalbumin, do not reflect naturally occurring situations in patients and can therefore only be used for proof of principle. Strong validation of these results is required in non-ovalbumin expressing preferably spontaneous tumour models in order to warrant clinical translation. Poly I:C has also been extensively tested in both animal models and patient studies and was shown to stimulate CD103^+^ tumour-infiltrating DCs, resulting in the induction of stronger anti-tumour effects and synergising with ICIs [48,70]. Another method being investigated to trigger immune responses uses activators of stimulators of interferon genes (STING) in DCs. Activation of STING leads to an increased secretion of type-I interferons, which facilitates cross-priming by DCs and are associated with a positive clinical response (Figure 1B) [71]. STING agonists have been shown to inhibit the growth of a variety of murine tumour models when injected intratumourally [72]. Moreover, Foote et al. combined treatment of a STING agonist, ADU-S100, with OX40 receptor activation and PD-L1 blockade to improve responses in mice that were not tolerant to the HER2 expressing murine breast cancer model NT2.5 [73].

Other methods used to activate TADCs and other antigen presenting cells (APCs) in vivo involve the use of mAbs targeting specific receptors expressed on these cells, or the delivery of mRNA coding for their ligands. APCs constitutively express the CD40 receptor from the TNF superfamily of receptors [74]. Ligation of CD40 by CD40L has been shown to alter the phenotype of human DCs, increasing their IL-12 secretion and boosting their ability to activate T cells [75]. Using CD40, agonist mAbs can mimic the responses induced by CD40L, making them interesting targets in the tumour context (Figure 1C). A phase I trial of a CD40 agonist combined with anti-CTLA-4 treatment was run for two years with 22 metastatic melanoma patients, of which nine of the treated patients were long-term survivors (survival of >3 years) [76]. The treatment was associated with evidence pointing towards increased T-cell activation and tumour infiltration. CD40 agonist treatment was also combined with radiotherapy, anti-CTLA-4, and anti-PD-1 treatments to generate long-term protection in a murine pancreatic ductal adenocarcinoma model [77]. The authors postulated that the combination of radiotherapy and CD40 agonism was able to disrupt and re-organise the links between the innate and adaptive immune systems in a non-redundant way, hence resulting in tumours that were re-sensitised to anti-PD-1 and anti-CTLA-4 therapy. CD40 ligation has also been incorporated in an mRNA-based adjuvant, referred to as TriMix mRNA (Figure 1D). TriMix mRNA contains three mRNA molecules, one coding for CD40 ligand, one for the co-stimulatory molecule CD70 and one for a constitutively activated TLR4. Administration of TriMix to tumour-bearing mice has been shown to mainly activate cDC1s leading to anti-tumour CTL responses [78]. Intratumoural administration of DCs electroporated in vitro with TriMix mRNA and melanoma antigens resulted in durable clinical benefits in patients with advanced melanoma in combination with anti-CTLA-4 mAb [79,80].

Another member of the TNF superfamily that has been shown to alter the TADC phenotype is CD137 (also referred to as 4-1BB or TNFRSF9), a co-stimulatory molecule expressed on murine DCs during their maturation. Triggering of CD137 using an agonistic mAb to CD137, was shown to increase IL-6 and IL-12 secretion as well as co-stimulatory molecule expression in vitro (Figure 1E) [81]. Depletion of CD11c^+^ cells using the diphtheria toxin system in the murine lymphoma model EG7-OVA reduced antigen cross-presentation to CTLs and subsequently inhibited anti-CD137-mediated EG7-OVA tumour eradication [82]. The reverse signalling action of CD137L was shown to suppress intratumoural differentiation of CD103^+^ DCs that secrete IL-12 [83]. When CD137L was blocked, IL-12 and IFNγ levels increased, restoring the CTL-driven anti-tumoural phenotype. CD103^+^ DCs in the tumour have also been reported to express higher levels of T cell immunoglobulin and mucin-domain containing protein-3 (TIM-3) in MMTV-PyMT murine mammary tumours, as well as human breast and mammary carcinoma. Inhibition of TIM-3 using an anti-TIM-3 mAb increased the secretion of CXCL9 by the CD103^+^ DCs, which led to an increased infiltration and activation of CTLs into MMTV-PyMT tumours, thereby improving the therapeutic activity of chemotherapeutic agent paclitaxel (Figure 1F) [84].

Just like many immune cells, DCs react when they sense danger signals. As such, any treatment that causes immunogenic cell death has the potential to promote DC maturation and to trigger immune responses (Figure 1G) [85,86]. As such, 65 °C radiofrequency ablation (RFA) of the murine EG7 lymphoma model was shown to trigger EG7 tumour cell death by necrosis. TADCs that phagocytosed 65 °C-treated EG7 cells expressed higher levels of MHCII and CD80 and were able to induce CTL responses, leading to the regression of small tumours (approx. 100 mm^3^) [87]. DC maturation was more enhanced after administration of CpG following RFA, resulting in the control of larger tumours and metastasis formation [87]. Combined radiotherapy, intratumoural CpG and PD-1 blockade reduced tumour growth and improved host survival using the murine lung cancer model LLC [88]. The tumours of mice treated with this triple combination therapy had increased frequencies of mature DCs and IFNγ^+^ and TNFα^+^ CTLs.

Saponin-based adjuvants have also been shown to mediate strong immune responses through the activation of both Th1 and CTL subsets [89]. These adjuvants were demonstrated to have the ability to activate the NLRP3 inflammasome, inducing IL-1β production in murine DC populations (Figure 1H) [90]. Upon subcutaneous injection of a saponin-based adjuvant, activated innate and adaptive immune cells were recruited to the draining LN, with CD8^+^ DCs displaying an enhanced maturation and an IL-12 secretory phenotype [91]. When a saponin-based adjuvant was combined with in situ tumour cryo-ablation, long-term anti-tumour immune memory responses were induced in mice [89]. After cryo-ablation of the primary B16 melanoma tumour, mice were re-challenged with B16 cells. 71% and 79% of mice treated with either of the two saponin-based adjuvants during the primary tumour ablation survived the secondary challenge, compared to 24% of mice that received no adjuvant with the cryo-ablation [87].

## 5. Antigen Delivery: Steering the Immune Response

The aim of DC-based therapies is to create anti-tumour CTL responses that can clear the tumour mass. Therefore, besides increasing the abundance of TADCs as well as their activation state, the durability of the anti-tumour response should be ensured. There are numerous DC-vaccination strategies described in literature, some of which are currently in clinical trials. However, based on a meta-analysis of cancer vaccine trials between 1991 and 2014, it appears that the number of clinical trials peaked in 2008, and has been declining ever since. Of these trials, very few observed results that were promising enough to warrant advancing from phase II to phase III [92]. Currently, only one DC-based treatment has been approved by the FDA, namely Sipuleucel-T. This treatment provided a 4.1-month overall survival increase for prostate cancer patients [93]. However, there are still DC-vaccination-based trials that have seen interesting results in long-term follow up. One phase II trial assessed the use of injecting Mo-DCs loaded with tumour lysate and matured by TNFα, Poly I:C, and IFNγ in 15 patients with surgically amenable liver metastasis of colon adenocarcinoma that underwent neoadjuvant chemotherapy, surgery and adjuvant chemotherapy. The vaccinated arm had a disease-free survival of 25.26 months, compared to 9.53 in the observation arm [94]. A phase III trial with 100 post-surgical lung cancer patients combined treatments of adjuvant chemotherapy and immunotherapy, whereby the immunotherapy comprised of autologous activated CTLs and DCs. The arm receiving the chemoimmunotherapy had a 2- and 5-year survival of 96% and 69.4%, respectively, compared to the chemotherapy control arm of 64.7% and 45.1%, respectively [95]. While not all of the trials report positive outcomes, nearly all of them indicate that DC-vaccination is safe and tolerable; however, much optimisation is still required to yield the most beneficial results. Nevertheless, with each new generation of DC vaccination, the treatments get more refined and sophisticated. New adjuvants, new antigens, new delivery systems, and new DC subsets are being investigated to provide DCs that can induce long-lasting anti-tumour immune responses in patients. The DC-based strategies currently in phase II clinical trials are listed in Table 1.

The success of DC vaccination largely depends on the target antigen. Currently, tumour-lysate based DC vaccines are believed to outperform tumour-associated antigen (TAA)/TAA-peptide pulsed DCs [96]. The mode of cell death in these vaccines also appears to influence the efficacy of the vaccine. Previous lysate preparations involved necrosis induced by freezing and thawing or mechanically induced necrosis [97]. More recently, oxidised tumour lysate appears to lead to better priming of T-cell responses [98,99]. Advances in technology have also made it possible to identify patient-specific mutations, opening the door to potential personalised neoantigen-based DC vaccination [5]. However, higher neoantigen loads are found in tumours with higher mutational burden, restricting the pool of potential patients to particular tumour types. Neoantigen vaccination also comes with the possible risk of inducing auto-immune reactions [100]. Common TAAs (MAGE, MLANA, gp100, WT1, NY-ESO-1) all still have a firm place in current DC vaccination, evidenced by their use in multiple current phase II trials (Table 1). It is unclear which type of antigen can provide the best in-patient results, especially as it is difficult to know exactly which neoantigen can induce strong T-cell responses. This being the case, it is likely that a combination of all applicable antigens would induce durable responses.

In contrast to the currently used blood-derived DC-based preparations, a strategy using tumour-derived cDC-based vaccines would not require ex vivo tumour-antigen loading, because these cells have naturally taken up tumour antigens. Indeed, tumour-derived cDC1s and cDC2s could confer protection in B16-OVA melanoma and LLC-OVA lung carcinoma tumours, respectively, when used as a prophylactic vaccination [13]. Tumour-derived cDCs could possibly be used to treat patients with unknown private or neo-tumour antigens, overcoming the need to identify them for therapeutic vaccination strategies. However, therapeutic effects should be shown in cancer models that do not express an artificial surrogate tumour antigen in order to warrant further trials. Another option to harness DCs in situ is by releasing antigens after tumour ablation, as exemplified in the previously mentioned study by the den Brok et al., in which in situ tumour destruction combined with saponin-based adjuvants stimulated TADCs to induce an anti-tumoural response that could protect mice after tumour re-challenge. This approach also forms a powerful in situ DC vaccine for which no prior knowledge of tumour antigens is required [89].

Tumour-associated antigens can also be directed towards DCs in vivo to induce immune responses. The use of antigens coupled to mAbs or to nanoparticles specific for DC receptors can be a way of directing antigen delivery specifically to DCs. Using the artificial tumour antigen ovalbumin, a group recently showed that it was possible to specifically deliver the ovalbumin antigen when bound to a nanoparticle specific for the complement C3 receptor that is expressed by APCs. The treatment triggered T-cell responses that were able to control a murine lymphoma model that expressed the ovalbumin tumour antigen [101]. Another DC-based vaccination method involves culturing autologous blood-derived DCs with known tumour-associated antigens for only 16 hours. Subsequently, these DCs were injected into patients where 4 of the 14 treated patients showed long-term progression-free survival between 12–35 months [102]. Three of these four patients developed CTL responses with high CD107a expression, as well as IFNγ, TNFα, and CCL4 production. Another way to deliver antigen specifically to DCs is by systemic intravenous administration of RNA-LPX. RNA-LPX encoding for a viral or mutant neoantigen can induce strong memory T-cell responses and lead to IFNγ-mediated tumour regression [103]. However, stimulating immune responses against one or more tumour antigens might not lead to long-term protection. Research from Saung et al. showed that mice treated with GVAX had the highest survival rates when treated with anti-PD-1 and anti-CSF1R inhibition, which altered the myeloid compartment of tumours in a model of murine pancreatic cancer [104]. Such combination therapies will likely form the future of DC-based cancer therapy. Several murine experiments on combinatorial treatments, in which at least one treatment is targeting DCs, show promising results. For instance, cDC2s were shown to be suppressed by regulatory T cells (Tregs) in the murine B16 tumour model. When Tregs were depleted using Foxp3DTR mice, the cDC2s were able to better activate CD4+ T cells in the tumour and generate anti-tumour responses [105]. Moreover, in an experiment combining lenalidomide and antigen-loaded DC vaccination, an enhanced anti-tumour response was observed in comparison to the corresponding monotherapies. Lenalidomide is an immunomodulatory drug shown to have antiangiogenic and -neoplastic properties. Besides, it also negatively influences Tregs by reducing their abundance and inhibiting their regulatory functions [106]. Early trials investigating potential successful combinations in humans are also underway. An in-situ vaccination strategy combining Flt3L, radiotherapy and poly-IC:LC was able to recruit activated, cross-presenting DCs to the tumour and increased a PD1^+^CD8^+^ T cell-population in non-responding patients. This same population that was increased in mice allowed them to become newly responsive to PD-1 blockade, which has resulted in a follow-up trial in humans [107]. Put together, these findings indicate that while achieving optimal DC responses is valuable, other factors must also be taken into account to induce optimal clinical effects.

## 6. Concluding Remarks

DC-based treatments have great potential in tackling cancer progression. However, DC-based treatments still require a lot of tinkering. The problems of low DC number, immature DC infiltration and antigen availability all need to be addressed. Advances in the understanding of DC biology and how DCs interact with cells infiltrating the TME, open new avenues to explore potential therapeutic interventions. Caution must be taken when new treatments are being translated from mouse to human, as what is true in mice is absolutely not guaranteed to work in humans. Treatments developed to increase DC abundance within tumours can be combined with adjuvants polarising these newly recruited TADCs away from a tolerogenic phenotype and towards a robust, CTL-stimulatory state. Most importantly, any potential DC-based cancer therapy also requires combination with other treatments. Therapies targeting the TME as well as other therapies such as chemotherapy, radiotherapy, ICIs, and tyrosine kinase inhibitors all have important roles to play in cancer treatment. Tumours are capable of exerting their immune evasion mechanisms by exploiting multiple different pathways, and any future treatment should mirror this and fight cancer through the concerted actions of multiple pathways. It is with this in mind that improvements can also be made in the way treatments are evaluated within clinical trials. While survival is still the most important factor to measure, perhaps monitoring the changes to levels of important immune biomarkers can be used as another definition of success. Treatment of terminal patients with DC-based treatments may not be enough to change the course of their disease, sheerly due to the level of progression. However, that same treatment may be incredibly effective in early-stage patients.

## Figures and Tables

**Table 1:** List of current DC trials phase II involving DCs.

ClinicalTrials.Gov Identifier	Title of Clinical Trial	DC Used	Antigen Loaded	Concurrent Treatments	Treated Cancer	Phase
NCT03410732	Dendritic Cell-based Immunotherapy in Treatment Gastric Cancer	Mo-DC	proteins from autologous tumour cell membrane	Post-radical surgery	Gastric Cancer	2
NCT03406715	Combination Immunotherapy-Ipilimumab-Nivolumab-Dendritic Cell p53 Vac-Patients With Small Cell Lung Cancer (SCLC)	Mo-DC	p53	Ipilimumab, Nivolumab	Small Cell Lung Cancer	2
NCT00703105	Ovarian Dendritic Cell Vaccine Trial	Mo-DC	Tumour lysate	ONTAK	Ovarian Cancer	2
NCT02033616	Autologous Dendritic Cells Loaded With Autologous Tumor Associated Antigens for Treatment of Advanced Epithelial Ovarian Carcinomas	Mo-DC	Autologous tumour associated antigens	-	Ovarian, fallopian and peritoneal cancer	2
NCT02718391	Complementary Vaccination With Dendritic Cells Pulsed With Autologous Tumor Lysate in Resected Stage III and IV Melanoma Patients. (ACDC)	Mo-DC	autologous tumour lysate or homogenate	post-surgical resection, IL-2	Melanoma	2
NCT01686334	Efficacy Study of Dendritic Cell Vaccination in Patients With Acute Myeloid Leukemia in Remission (WIDEA)	Mo-DC	Wilms’ tumour antigen	-	Acute Myeloid Leukemia	1, 2
NCT03610360	DENdritic Cell Immunotherapy for Mesothelioma (DENIM)	Mo-DC	Allogenic tumour cell lysate	-	Mesothelioma	2
NCT03697707	Efficacy and Safety of Immunotherapy With Allogeneic Dendritic Cells, DCP-001, in Patients With Acute Myeloid Leukaemia (ADVANCE-II)	Mo-DC	DCP-001	-	Acute Myeloid Leukaemia	2
NCT00338377	Lymphodepletion Plus Adoptive Cell Transfer With or Without Dendritic Cell Immunization in Patients With Metastatic Melanoma	Mo-DC	MART-1	Cyclophosphamide, Fludarabine, IL-2, T cells, Mesna	Melanoma	2
NCT02649582	Adjuvant Dendritic Cell-immunotherapy Plus Temozolomide in Glioblastoma Patients (ADDIT-GLIO)	Mo-DC	Wilms’ tumour antigen	Temozolmide	Glioblastoma	2
NCT03325101	Dendritic Cell Therapy After Cryosurgery in Combination With Pembrolizumab in Treating Patients With Stage III-IV Melanoma That Cannot Be Remove by Surgery	Mo-DC	-	Pembrolizumab	Melanoma	1b, 2
NCT02334735	A Comparison of Matured Dendritic Cells and Montanide® in Study Subjects With High Risk of Melanoma Recurrence	Mo-DC	KLH, NY-ESO-1, MART-1	Poly-ICLC	Melanoma	2
NCT02709616	Personalized Cellular Vaccine for Glioblastoma (PERCELLVAC) (PERCELLVAC)	Mo-DC	tumour antigen mRNA	-	Glioblastoma	1, 2
NCT01976585	In Situ Vaccine for Low-Grade Lymphoma: Combination of Intratumoral Flt3L and Poly-ICLC With Low-Dose Radiotherapy	Tumoural DCs	-	Flt3L, Poly-ICLC	Lymphoma	2
NCT01983748	Dendritic Cells Plus Autologous Tumor RNA in Uveal Melanoma	Mo-DC	Autologous tumour RNA	-	Uveal melanoma	3
NCT02993315	Melanoma Patients Immunized With Natural DenDritic Cells (MIND-DC)	mDC and pDC	antigen-loaded	-	Melanoma	3, 4
